# Association between body roundness index and osteoarthritis: a cross-sectional analysis of NHANES 2011–2018

**DOI:** 10.3389/fnut.2024.1501722

**Published:** 2024-10-31

**Authors:** Huazheng Liang, Wenyue Si, Lin Li, Kaiying Yang

**Affiliations:** ^1^Department of Pediatric Surgery, Guangzhou Women and Children's Medical Center, National Children's Medical Center for South Central Region, Guangzhou Medical University, Guangzhou, China; ^2^School of Pediatrics, Guangzhou Medical University, Guangzhou, China; ^3^Department of Science Research and Education Management, Guangzhou Women and Children's Medical Center, National Children's Medical Center for South Central Region, Guangzhou Medical University, Guangzhou, China; ^4^Department of Pediatric Surgery, Guangdong Provincial Key Laboratory of Research in Structural Birth Defect Disease, Guangzhou Women and Children's Medical Center, Guangzhou Medical University, Guangdong Provincial Clinical Research Center for Child Health, Guangzhou, China

**Keywords:** osteoarthritis, body roundness index, obesity, cross-sectional study, National Health and Nutrition Examination Survey

## Abstract

**Objective:**

The objective of this study was to investigate the potential association between body roundness index (BRI) and the risk of osteoarthritis (OA) in US adults.

**Methods:**

A cross-sectional analysis consisting of 20,232 participants was conducted using data from the National Health and Nutrition Examination Survey (NHANES) from 2011 to 2018. Participants (≥20 years of age) were included and divided into OA and non-OA groups. Then, the demographics and characteristics of the participants were compared between the two groups. The relationship between BRI and OA was assessed using a multivariate logistic regression model with fitted smoothed curve techniques. Additionally, subgroup analyses on the correlation between BRI and OA were performed.

**Results:**

The BRI scores in OA group were significantly higher than in the non-OA group (6.60 ± 2.62 vs. 5.46 ± 2.34, *p* < 0.001). Multivariate logistic analysis revealed that a significantly positive association between BRI and OA (OR = 1.12, 95% CI: 1.09–1.14, *p* < 0.001). In the subgroup analysis, only the race subgroup showed a significant difference between BRI and OA (*p* < 0.001).

**Conclusion:**

Our findings highlight a significantly positive association between BRI and OA prevalence in the general US population.

## 1 Introduction

Osteoarthritis (OA), a common chronic joint disease, is characterized by the progressive deterioration of articular cartilage, the formation of secondary osteophytes, and adaptive changes in subchondral bone (1, 2). Recently, a study by Hunter et al. in 2020 reported that OA affects approximately 3.8% of the global population, equating to approximately 250 million people (3). The incidence and prevalence of OA are estimated to increase significantly in the coming decades, primarily due to an aging population, increasing obesity rates, and a high incidence of traumatic knee injuries (4). OA represents a significant burden on global public health, causing widespread pain, severely limiting daily activities, and in some cases leading to disability, all of which significantly affect socioeconomic status and quality of life (5). Nevertheless, the etiology of OA remains unclear, and its risk factors are complex, including demographic-level factors such as age, sex, and obesity, as well as joint-level factors such as injury and malalignment (6). Therefore, determining modifiable risk factors may contribute to the development of strategies aimed at delaying OA progression and reducing its burden on public health.

Recently, an increasing number of scientific studies have indicated a strong association between obesity and the development of OA. Obesity may affect the development of OA by altering metabolism and inflammation in the body (7, 8). Obesity is a chronic and complex disease characterized by excessive fat deposition, which can lead to health damage. It can be diagnosed by measuring body mass index (BMI) or waist circumference (9, 10). However, the use of BMI and waist circumference to diagnose obesity has significant limitations. BMI does not account for the specifics of an individual’s fat distribution and is subject to significant racial and individual differences, which may lead to misclassification of obesity (11, 12). While the waist circumference index reflects abdominal fat accumulation to some extent, it also fails to reveal the full extent of body fat distribution (13). In contrast, the body roundness index (BRI), as introduced by Thomas et al., is an emerging assessment tool that offers a more comprehensive evaluation of body fat distribution (14). By considering the ratio of body width to body height, the BRI provides a more detailed assessment of an individual’s body shape, including the distribution of adiposity throughout the body, allowing it to more accurately reflect obesity status and its potential health risks (14).

To date, an increasing number of studies have indicated a correlation between the BRI and various health conditions, including cardiovascular disease, depression and diabetes (15–19). However, no studies have investigated the potential association between the BRI and OA. Therefore, the present study was conducted to explore the potential association between the BRI and OA among U.S. adults via data from the National Health and Nutrition Examination Survey (NHANES). In addition, our study elucidates the impact of obesity patterns on the risk of developing OA and facilitates the development of targeted prevention and treatment strategies.

## 2 Materials and methods

### 2.1 Data sources and study participants

The NHANES is a comprehensive, population-based survey research project sponsored by the National Center for Health Statistics (20, 21). The survey incorporates a detailed methodology involving interviews, physical examinations, and laboratory tests designed to assess the health and nutritional status of adults and children in the United States (22, 23). The data collection process for NHANES adheres to the highest ethical standards, with all study procedures approved by the National Research Ethics Board (1). Furthermore, informed consent was obtained from all participants. The detailed study design and data from NHANES are publicly available for review at: https://www.cdc.gov/nchs/nhanes/.

**Figure 1:**
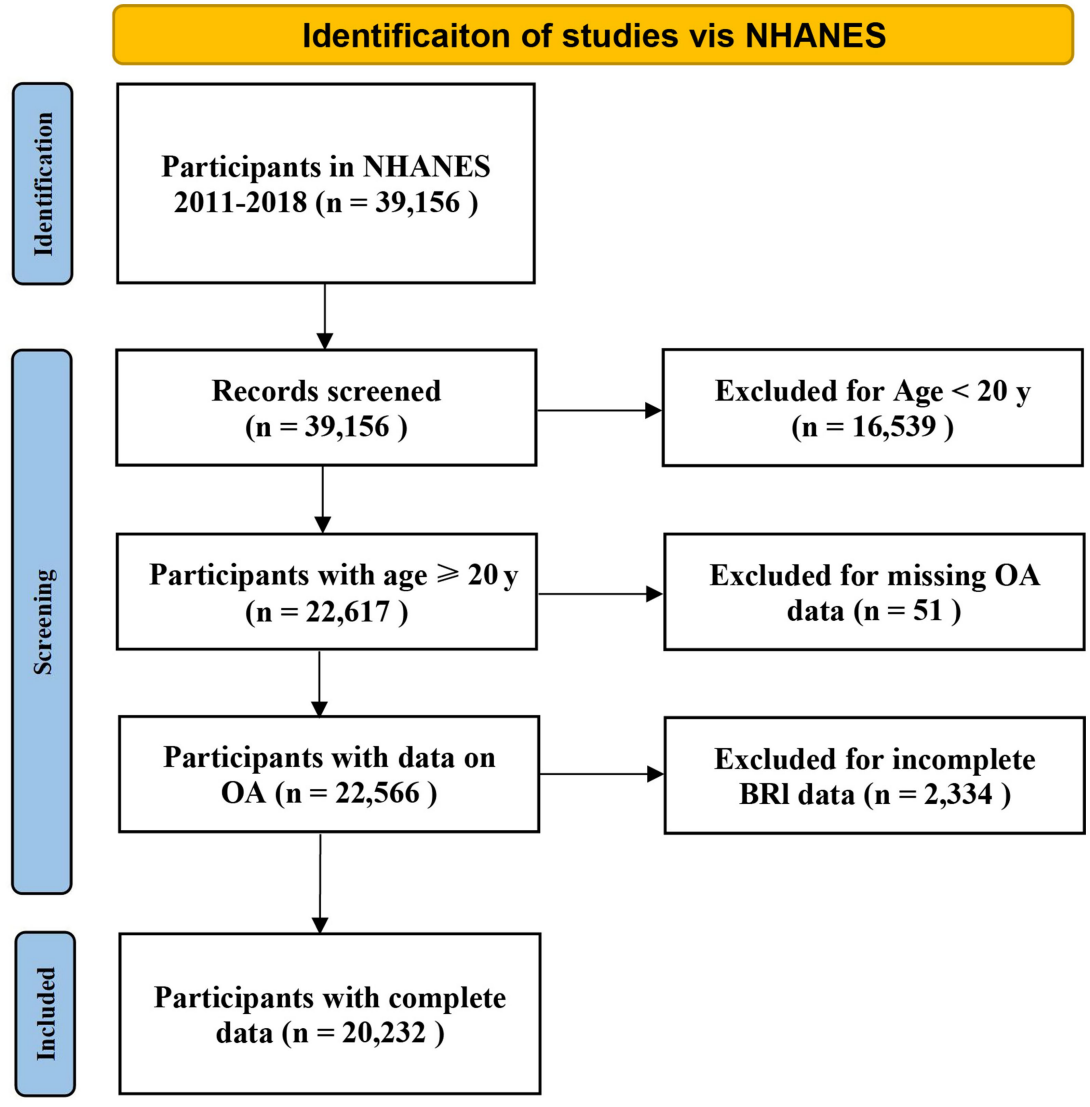
Flow chart demonstrating the exclusion and inclusion criteria for identifying OA patients from NHANES 2011-2018. NHANES, National Health and Nutrition Examination Survey.

The data used in this study were derived from the NHANES database, which covers the results collected from four consecutive survey cycles between 2011 and 2018. A total of 39,156 participants were included in the study. Participants younger than 20 years of age (*n* = 16,539) were excluded. Additionally, those with missing OA (*n* = 51) or BRI (*n* = 2,334) data were also excluded. A total of 20,232 individuals participated in this study (Figure 1).

### 2.2 Assessment of OA and BRI

A previous study reported up to 81% agreement between self-reported and clinically validated outcomes of OA (24). In the NHANES database, OA status is primarily determined on the basis of a self-report questionnaire collected under the category of “medical conditions.” Participants were first asked, “Has a doctor or other health professional ever told you that you had arthritis?.” Those who answered “no” were excluded from further analysis in our study. If the response was positive, the participants were then asked the following question: “Which type of arthritis was it?.” Those who self-reported “osteoarthritis” were defined as patients with OA, whereas those who reported having a different form of arthritis, including “rheumatoid arthritis and psoriatic arthritis,” and their failure to answer the question were categorized as non-OA patients. Previous studies have supported the validity of self-reported OA concerns (25–28). Each NHANES participant was required to undergo a physical examination, which included measurements of height and waist circumference. The BRI formula, developed by Thomas et al., is calculated as *364.2–365.5 × (1 – [(waist circumference (m)/2π)^2^/(0.5 × height(m))^2^])^½^* (14).

### 2.3 Covariates

When exploring the association between the BRI and OA, it is important to consider the potential influence and moderating effects of multiple covariates on this relationship. Therefore, we included a total of 14 confounders in this study. The demographic data included age (≥20 years), sex (male vs. female), race (Mexican American, non-Hispanic white, non-Hispanic black, other Hispanic and other races including multiracial), education level (under high school, high school or equivalent, and above high school), marital status (married/cohabiting, widowed/divorced/separated, never married), and family income–poverty ratio (PIR), classified into three categories (< 1.3, 1.3–3.49, and ≥ 3.5), which are distinct segments rather than tertiles as determined by relevant NHANES studies (29, 30). The examination data included weight and BMI, which were classified as low to normal weight (<25), overweight (25–29.9), or obese (≥30) (31). The questionnaire section included questions concerning participation in moderate recreational activities (yes/no), diabetes (yes/no), hypertension (yes/no), cancer (yes/no), and alcohol use. Alcohol use and cigarette use were also assessed. Alcohol use was defined on the basis of the average daily consumption of alcoholic beverages in the past 12 months (32). Cigarette use was differentiated between nonsmokers (total lifetime number of cigarettes <100 cigarettes) and smokers (total lifetime number of cigarettes ≥100 cigarettes) on the basis of lifetime number of cigarettes smoked (33).

### 2.4 Statistical analysis

Continuous variables are expressed as the means with standard deviations (SDs), whereas categorical variables are expressed as percentages. The Kruskal–Wallis test was employed for continuous variables, and the χ^2^ test was used for categorical variables. In this study, multiple logistic regression analyses models were used to explore the correlation between the BRI scores and OA. The multiple logistic regression analysis was divided into three models: Model 1 was unadjusted; Model 2 was adjusted for age, sex, and race; and Model 3 was fully adjusted for age, sex, race, PIR, cigarette use, hypertension, education, diabetes, cancer, marital status, moderate recreational activities, and alcohol use. Furthermore, the smoothed curve fitting methods using a generalized additive model (GAM) were used to investigate the nonlinear association between the BRI index and OA. To further elucidate the potential influence of different subgroups on the correlation between BRI and OA, an interaction test was conducted to assess the impact of pertinent clinical confounders. All data analyses were conducted via Empower Stats software, version 2.0^1^ (X&Y Solutions, Inc., Boston, MA). A two-sided *p* value of less than 0.05 was considered statistically significant.

## 3 Results

### 3.1 Baseline characteristics of the participants

The detailed baseline characteristics of the participants with and without OA are presented in Table 1. A total of 20,232 participants were enrolled in the present study, with a mean age of 49 years, of whom 48.75% were male and 51.25% were female. Of the 20,232 participants, 2,279 were reported to have OA. Age, sex, race, education, marital status, PIR, BMI, diabetes, cigarette use, alcohol use, hypertension, cancer, height, weight, waist circumference, and BRI were significantly different between the non-OA and OA groups. Compared with the non-OA group, the OA group had a greater proportion of women (51.25% vs. 48.75%), more never-married people (78.21% vs. 21.79%), and more people with a BMI of 25 or higher (71.04% vs. 28.96%). Moreover, OA is associated with higher levels of education, PIRs, and BRIs, among others.

**Table 1:** The baseline characteristics of participants stratified by self-reported osteoarthritis (OA) among U.S. adults from the NHANES 2011–2018.

Characteristic	Total *N* = 20,232 (100%)	Non-OA *N* = 17,953 (88.74%)	OA *N* = 2,279 (11.26%)	*p*-value
Age (year)				< 0.001^a^
20 ~ 39	6,872 (3.97%)	6,728 (37.48%)	144 (6.32%)	
40 ~ 59	6,775 (33.49%)	6,117 (34.07%)	658 (28.87%)	
≥60	6,585 (32.55%)	5,108 (28.45%)	1,477 (64.81%)	
Sex, *n* (%)				< 0.001^a^
Male	9,863 (48.75%)	9,040 (50.35%)	823 (36.11%)	
Female	1,036 (51.25%)	8,913 (49.65%)	1,456 (63.89%)	
Race, *n* (%)				< 0.001^a^
Mexican American	2,740 (13.54%)	2,555 (14.23%)	185 (8.12%)	
Non-Hispanic White	7,449 (36.82%)	6,143 (34.22%)	1,306 (57.31%)	
Non-Hispanic Black	4,591 (22.69%)	4,202 (23.41%)	389 (17.07%)	
Other Hispanic	2,126 (10.51%)	1950 (10.86%)	176 (7.72%)	
Other race/multiracial	3,326 (16.44%)	3,103 (17.28%)	223 (9.78%)	
Education, *n* (%)				< 0.001^a^
Under high school	4,394 (21.72%)	3,969 (22.11%)	425 (18.65%)	
High School or equivalent	4,495 (22.22%)	3,992 (22.24%)	503 (22.07%)	
Above high school	11,343 (56.06%)	9,992 (55.66%)	1,351 (59.28%)	
Marital status, *n* (%)				< 0.001^a^
Married/cohabiting	3,925 (19.40%)	3,733 (20.79%)	192 (8.42%)	
Widowed/divorced/separated	12,000 (59.31%)	10,662 (59.39%)	1,338 (58.71%)	
Never married	4,307 (21.29%)	3,558 (19.82%)	749 (32.87%)	
Moderate recreational activities, *n* (%)				< 0.001^a^
Yes	8,459 (41.81%)	7,615 (42.42%)	844 (37.03%)	
No	11,773 (58.19%)	10,338 (57.58%)	1,435 (62.97%)	
PIR (%)				< 0.001^a^
<1.30	5,933 (29.32%)	5,351 (29.81%)	582 (25.54%)	
1.30 ~ 3.49	8,754 (43.27%)	7,755 (43.20%)	999 (43.84%)	
≥3.50	5,545 (27.41%)	4,847 (27.00%)	698 (30.63%)	
Alcohol use, *n* (%)	20,232 (3.79)	3.68 ± 23.12	4.70 ± 42.80	< 0.001^a^
Cigarette Use, *n* (%)				< 0.001^a^
Smoked ≥ 100 cigarettes in life	8,631 (42.66%)	7,444 (41.46%)	1,187 (52.08%)	
Smoked < 100 cigarettes in life	11,601 (57.34%)	10,509 (58.54%)	1,092 (47.92%)	
Diabetes, *n* (%)				< 0.001^a^
Yes	2,731 (13.50%)	2,212 (12.32%)	519 (22.77%)	
No	17,501 (86.50%)	15,741 (87.68%)	1760 (77.23%)	
Hypertension, *n* (%)				< 0.001^a^
Yes	7,348 (36.32%)	5,945 (33.11%)	1,403 (61.56%)	
No	12,884 (63.68%)	12,008 (66.89%)	876 (38.44%)	
Cancer, *n* (%)				< 0.001^a^
Yes	1873 (9.26%)	1,407 (7.84%)	466 (20.45%)	
No	18,359 (90.74%)	16,546 (92.16%)	1813 (79.55%)	
Weight (kg)	20,232 (81.59)	81.12 ± 21.32	85.27 ± 23.35	<0.001^a^
Height (cm)	20,232 (166.81)	167.05 ± 10.10	164.94 ± 10.08	< 0.001^a^
BMI (%)				< 0.001^a^
<25	5,860 (28.96%)	5,399 (30.07%)	461 (20.23%)	
25 ~ 29.9	6,532 (32.29%)	5,864 (32.66%)	668 (29.31%)	
≥30	7,840 (38.75%)	6,690 (37.26%)	1,150 (50.46%)	
Waist circumference (cm)	20,232 (99.61)	98.85 ± 16.44	105.60 ± 17.06	< 0.001^b^
BRI	20,232 (5.59)	5.46 ± 2.34	6.60 ± 2.62	< 0.001^b^

Continuous variables are presented as the means ± standard deviations. Categorical variables are presented as *n* (%). ^a^Chi-square test, ^b^Kruskal–Wallis rank sum test. OA, osteoarthritis; Non-OA, nonosteoarthritis; BMI, body mass index; BRI, body roundness index; PIR, family income–poverty ratio.

### 3.2 Associations between the BRI and the risk of OA

To investigate the relationship between BRI and OA, a stepwise adjusted model using multivariate logistic regression was performed, as illustrated in Table 2. Our results revealed a positive correlation between the BRI and OA. The positive association remained in Model 3, even after adjusting for all relevant variables (OR = 1.12, 95% CI: 1.09–1.14), indicating that for each unit increase in the BRI, the odds of developing OA increased by 12%. For sensitivity analysis, the BRI was further transformed from a continuous variable into categorical quartiles (Table 2). The positive correlation between the BRI and OA remained consistent across all three adjusted models. According to the fully adjusted model, the increased odds of OA were significantly greater in the highest quartile than in the lowest quartile (OR = 1.86, 95% CI: 1.59–2.18).

**Table 2:** Associations between BRI scores and its quartiles and the risk of OA in participants.

	OR (95% CI), *p*-value		
	Model 1	Model 2	Model 3
	(*N* = 20,232)	(*N* = 20,232)	(*N* = 20,232)
BRI	1.19 (1.17, 1.20), < 0.001	1.14 (1.12, 1.16), < 0.001	1.12 (1.09, 1.14), < 0.001
Stratified by BRI quartiles			
Q1	Reference	Reference	Reference
Q2	1.90 (1.63, 2.21), < 0.001	1.32 (1.12, 1.55), < 0.001	1.24 (1.06, 1.46), 0.009
Q3	2.40 (2.07, 2.79), < 0.001	1.45 (1.24, 1.70), < 0.001	1.30 (1.11, 1.53), 0.001
Q4	3.80 (3.30, 4.38), < 0.001	2.18 (1.87, 2.54), < 0.001	1.86 (1.59, 2.18), < 0.001
*P* for trend	< 0.001	< 0.001	< 0.001

Model 1: adjusted for none. Model 2: adjusted for age (years), sex, and race. Model 3: adjusted for age (years), sex, race, PIR, cigarette use, hypertension, education, diabetes, cancer, marital status, moderate recreational activities, and alcohol use. BMI, body mass index; PIR, family income–poverty ratio; OR, odds ratio; CI, confidence interval.

### 3.3 Nonlinear relationship between the BRI and OA

**Figure 2:**
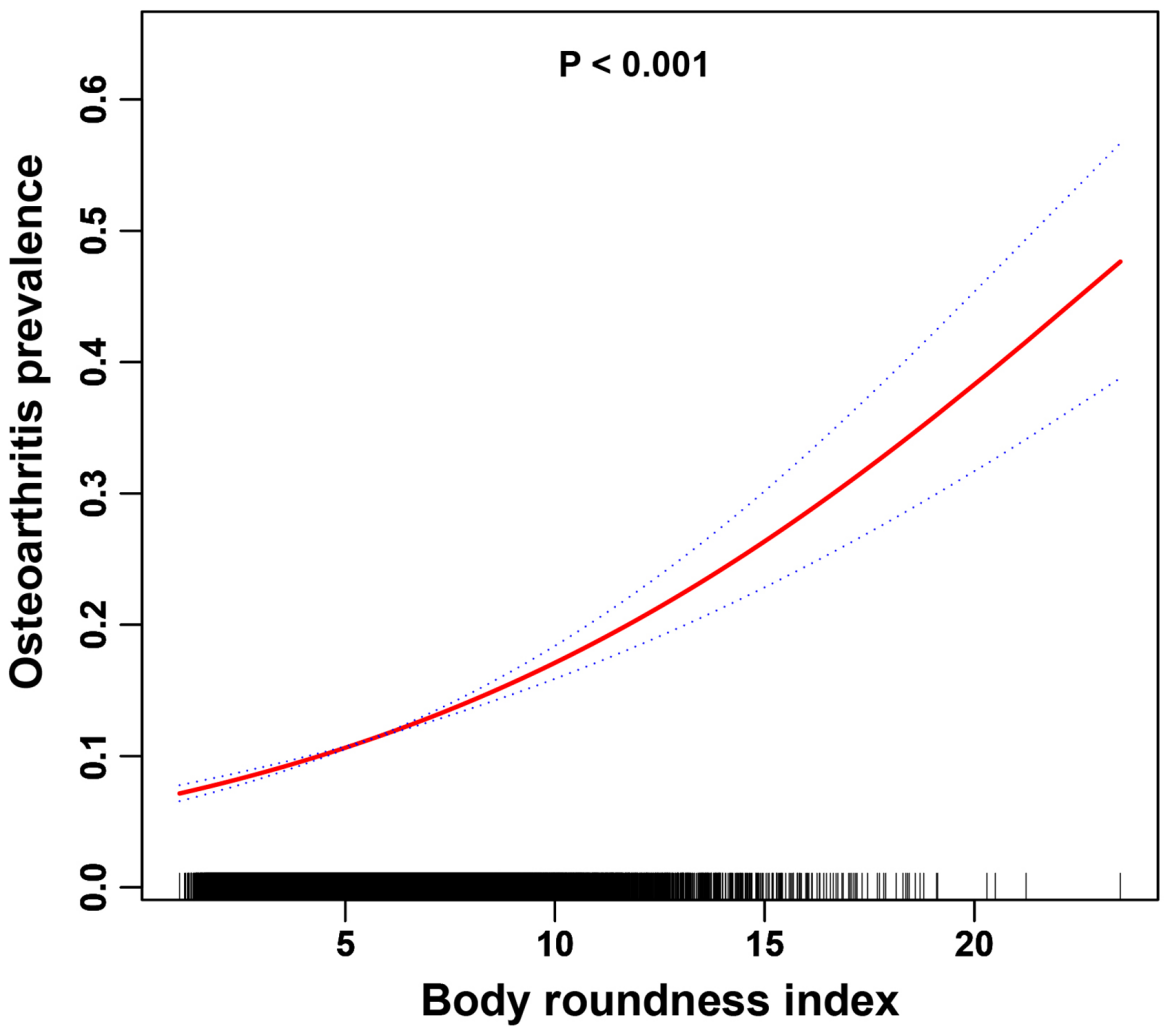
The association between the BRI index and OA is illustrated via smoothed curve fitting methods using a generalized additive model. Adjusted for age, sex, race, PIR, cigarette use, hypertension, education, diabetes, cancer, marital status, moderate recreational activities, and alcohol use. The solid red line depicts the smooth curve fit between the variables, whereas the blue bands represent the 95% confidence intervals derived from the fit.

**Figure 3:**
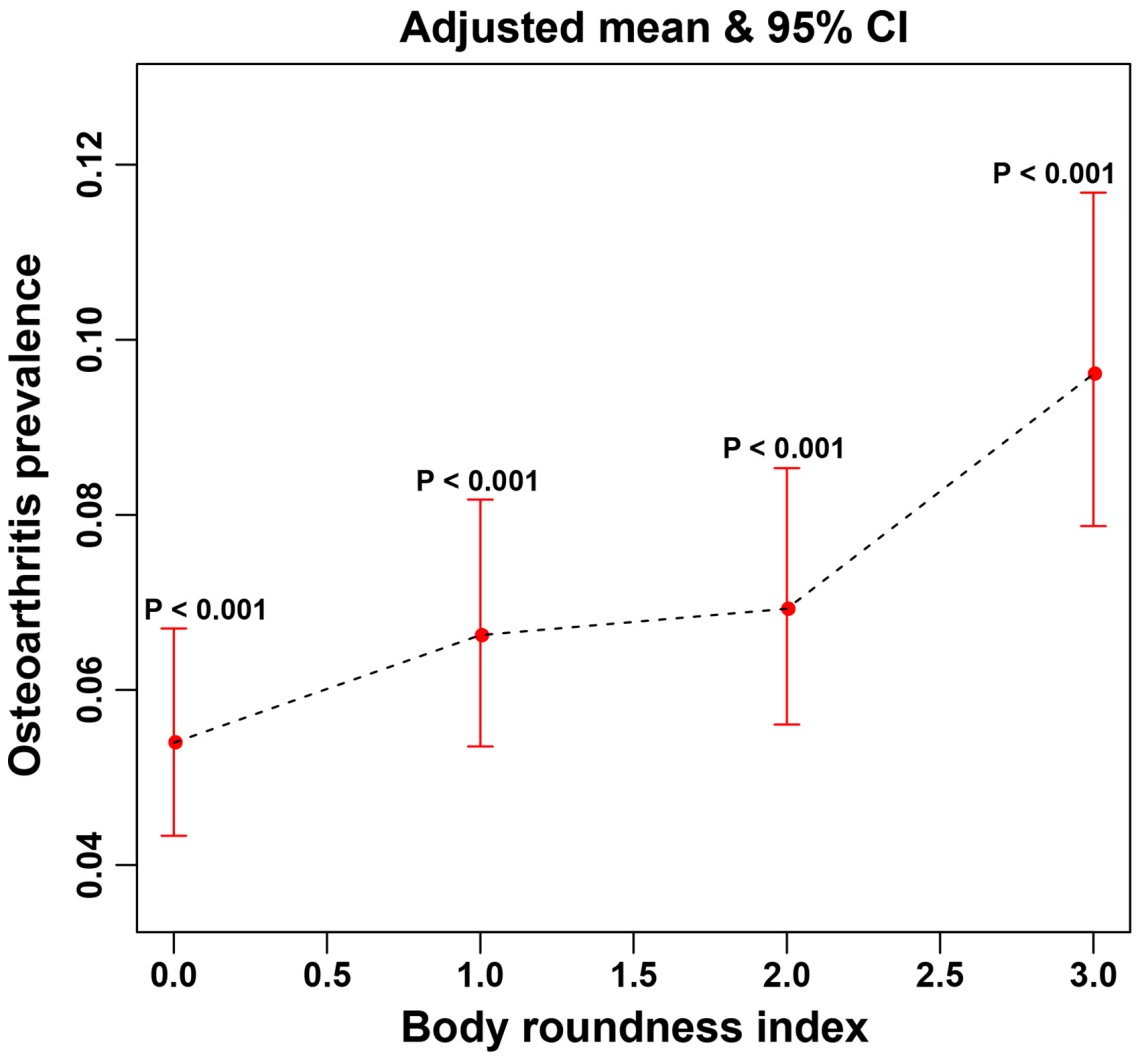
The associations between quartiles of BRI and OA were analyzed via smoothed curve fitting. The x-axis represents the quartiles of the BRI, which are categorized as Q1, Q2, Q3, and Q4, with the corresponding values indicated on the axis. The y-axis displays the adjusted mean prevalence of OA and the 95% confidence intervals for each BRI quartile. *P*-interaction was used to evaluate the interaction, with <0.05 indicating statistical significance.

In this study, the use of smoothed curve fitting facilitated the investigation of the nonlinear relationship between the BRI index and its tangent into quartiles and OA. The findings of the study indicated a nonlinear, positive correlation between BRI levels and OA (Figures 2, 3).

### 3.4 Subgroup analysis

In this study, a subgroup analysis was conducted to examine the stability of the nonlinear positive association between the BRI and OA across the identified subgroups (Table 3). The results of subgroup analyses revealed that a significant difference in the correlation between BRI and OA across different race groups (*P* for interaction <0.0001). For race subgroup, the OR (95%CI) of Mexican American, non-Hispanic White, non-Hispanic Black, other Hispanic and other races/multiracial were 1.13 (1.06, 1.20), 1.15 (1.07, 1.23), 1.07 (1.05, 1.10), 1.14 (1.10, 1.18), and 1.27 (1.19, 1.35), respectively. However, the association between the BRI and OA was not influenced by sex, education level, cigarette use, marital status, moderate recreational activities, PIR, hypertension, cancer, or diabetes mellitus (all *P* for interaction >0.05). This finding indicates that the nonlinear positive correlation between the BRI and OA remained robustly stable across all the cases except subgroup of race.

**Table 3:** Subgroup analysis of the effect of the body roundness index on osteoarthritis (*N* = 20,232).

Subgroup	*N*	OR (95% CI)	*p*-value	*P* for interaction
Age (year)				0.075
20 ~ 39	6,872	1.14 (1.08, 1.20)	<0.001	
40 ~ 59	6,775	1.14 (1.10, 1.17)	<0.001	
≥60	6,585	1.09 (1.06, 1.12)	<0.001	
Sex				0.698
Male	9,863	1.12 (1.08, 1.16)	<0.001	
Female	10,369	1.11 (1.09, 1.14)	<0.001	
Race				< 0.001
Mexican American	2,740	1.13 (1.06, 1.20)	<0.001	
Non-Hispanic White	2,126	1.15 (1.07, 1.23)	<0.001	
Non-Hispanic Black	7,449	1.07 (1.05, 1.10)	<0.001	
Other Hispanic	4,591	1.14 (1.10, 1.18)	<0.001	
Other race/multiracial	3,326	1.27 (1.19, 1.35)	<0.001	
Education				0.675
Under high school	4,394	1.10 (1.05, 1.15)	<0.001	
High School or equivalent	4,495	1.11 (1.07, 1.16)	<0.001	
Above high school	11,343	1.12 (1.09, 1.15)	<0.001	
Marital status				0.281
Married/cohabiting	3,925	1.16 (1.10, 1.21)	<0.001	
Widowed/divorced/separated	12,000	1.11 (1.08, 1.14)	<0.001	
Never married	4,307	1.11 (1.07, 1.15)	<0.001	
Moderate recreational activities				0.394
Yes	8,459	1.10 (1.07, 1.14)	<0.001	
No	11,773	1.12 (1.10, 1.15)	<0.001	
PIR				0.863
<1.30	5,933	1.11 (1.07, 1.15)	<0.001	
1.30 ~ 3.49	8,754	1.12 (1.09, 1.15)	<0.001	
≥3.50	5,545	1.12 (1.08, 1.16)	<0.001	
Cigarette Use				0.131
Smoked ≥ 100 cigarettes in life	8,631	1.10 (1.07, 1.13)	<0.001	
Smoked < 100 cigarettes in life	11,601	1.13 (1.10, 1.16)	<0.001	
Diabetes				0.489
Yes	2,731	1.13 (1.08, 1.17)	<0.001	
No	17,501	1.11 (1.09, 1.14)	<0.001	
Hypertension				0.487
Yes	7,348	1.11 (1.08, 1.14)	<0.001	
No	12,884	1.12 (1.09, 1.16)	<0.001	
Cancer				0.513
Yes	1873	1.10 (1.05, 1.15)	<0.001	
No	18,359	1.12 (1.09, 1.14)	<0.001	

Age, sex, race, PIR, cigarette use, hypertension, education, diabetes status, cancer status, marital status, and moderate recreational activities were adjusted. *P* for interaction were used to evaluate whether the association between BRI and OA differed across various subgroups in the regression models, such as sex, education level, cigarette use, marital status, moderate recreational activities, PIR, hypertension, cancer, and diabetes mellitus. PIR, family income–poverty ratio.

## 4 Discussion

Obesity has been considered the key modifiable risk factor for OA (34). Recently, a considerable number of clinical studies have focused on investigating the relationships between various indicators of obesity (such as BMI, waist circumference, and muscle mass) and the development and progression of OA (34–36). An observational study by Zhang et al. demonstrated that an increase in BMI, a traditional measure of obesity, is positively associated with the risk of OA, with the effect being particularly significant in patients with knee OA (35). In addition, a regional study in the U.S. revealed that an increase in waist circumference, a measure of abdominal obesity, is not only associated with an elevated risk of OA but also may predict a future decline in physical function (36). This highlights the importance of monitoring waist circumference alongside BMI in the clinical management of OA. Moreover, interest in the relationship between the loss of muscle mass, known as sarcopenia, and OA is increasing. Several studies have suggested that sarcopenia may increase the risk of OA by affecting the body’s metabolic and functional status (37–39). These findings demonstrate the close association between obesity and OA. However, these traditional obesity indicators have their limitations. For instance, BMI does not account for individual fat distribution, and waist circumference only reflects abdominal obesity without capturing overall body fat distribution. Given these limitations, our study therefore explores the relationship between BRI and OA to provide a more comprehensive understanding of how visceral fat may influence OA development. This not only reinforces the link between obesity and OA but also provides a new direction for future research, highlighting the importance of body fat distribution in OA risk assessment.

Previously, substantial evidence indicated a significant correlation between the BRI and various diseases (18, 40–43). A cross-sectional study revealed that the BRI was associated with an estimated low glomerular filtration rate in a Chinese population, providing evidence that central obesity may play a role in the progression of chronic kidney disease (40). A retrospective study conducted in Japan demonstrated a positive and non-linear correlation between the BRI and diabetes mellitus incidence, indicating a threshold effect: when BRI exceeds 4.137 in females and 3.146 in males, a significant positive correlation with the incidence of type 2 diabetes mellitus is observed (41, 44). Additionally, a cross-sectional study using NHANES data indicated a strong, nonlinear relationship between BRI and metabolic syndrome (MetS), suggesting that the BRI may effectively identify individuals at risk for MetS (42). Furthermore, meta-analyses suggest a bidirectional association between MetS and OA, implying that OA can predict an increased risk of MetS, while MetS may also promote the development of OA (45). Moreover, BRI is shown to outperform traditional anthropometric indices, such as BMI, body shape index, and body adiposity index, in predicting MetS (46). In addition, BRI has been a positive association with hypertension and cardiovascular disease (47). Consequently, these studies implies that BRI may be associated with the OA risk through its association with MetS, thereby supporting its use as an indicator for assessing OA risk.

On the basis of the above research findings, we hypothesize that there may be an intrinsic association between the BRI and OA. To the best of our knowledge, this is the first cross-sectional study investigating the association between the BRI and OA. By analyzing cross-sectional data from 20,232 participants, we identified a significant and nonlinear positive association between the BRI and OA, providing compelling evidence for understanding the relationship between these two variables. Furthermore, through rigorous multivariate logistic regression analyses, we excluded the interference of other potential confounders and confirmed that a high BRI is an independent and significant risk factor for the development of OA. Subgroup analyses revealed that the associations between the BRI and OA were consistent across various factors, including sex, education level, cigarette use, marital status, moderate recreational activities, PIR, hypertension, cancer, and diabetes. Additionally, racial differences were found to partially affect the association between BRI and OA. Racial differences may influence the onset and manifestation of OA, potentially through a combination of biological and social factors, including genetic susceptibility, environmental factors, and lifestyle (48). Moreover, the significant differences in radiological, symptomatic, and clinical manifestations of hand OA between races highlight the potential role of race-specific biological mechanisms and environmental risk factors in the pathogenesis of osteoarthritis (49). These findings offer new insight into clinical risk prediction, suggesting that early identification of individuals with high BRI and targeted interventions could slow the progression of OA and improve patient quality of life.

The BRI, a recently developed indicator for assessing the degree of obesity in humans, may be associated with the development of OA through a variety of mechanisms. A review by Sampath et al. emphasized the strong correlation between obesity, metabolic syndrome, and OA (50). Specifically, obesity induces a chronic low-grade inflammatory state, which can accelerate cartilage degradation. This process is mediated by elevated levels of different mediators, especially those with proinflammatory qualities, such as interleukin-1β (IL-1β), interleukin-6 (IL-6), and tumor necrosis factor-*α* (TNF-α), thereby increasing the risk of developing OA. Moreover, individuals with obesity typically exhibit elevated levels of adipokines, which play pivotal roles in regulating energy metabolism, inflammation, and the immune response. Research by Xie and Chen investigated the potential of adipokines as novel therapeutic targets in osteoarthritis (OA) (51). Specific adipokines, such as leptin, adiponectin, and resistin, are expressed at abnormal levels in the joint fluid and serum of patients with OA, indicating that they may play critical roles in the pathological process of OA. For example, leptin and resistin have been shown to exert proinflammatory effects and enhance the expression of proinflammatory cytokines such as IL-1β and TNF-*α*. This, in turn, contributes to an increased risk of developing OA. In addition, Zhu and colleagues demonstrated that increasing Sirt3 expression in mice reduced OA lesions induced by a high-fat diet (52). These findings suggest that Sirt3 may play a role in protecting cartilage and slowing OA progression. Furthermore, the symptoms associated with decreased Sirt3 expression levels in individuals on high-fat diets could provide a potential mechanism for the observed positive correlation between BRI and OA. These studies suggest a potential mechanism by which the BRI may contribute to the development of OA.

This study has several strengths. First, this is an innovative study revealing the association between the BRI and OA. In the present study, the BRI, an emerging assessment tool, was used instead of traditional obesity measurement indicators such as BMI or waist circumference to evaluate individual body characteristics more comprehensively. Second, the reliability and representativeness of our study were significantly enhanced by the large sample size collected from the NHANES database and the precise covariate correction measures. Additionally, the sensitivity analysis enabled us to effectively minimize the probability of false-positive results. However, this study also has several limitations. Specifically, the cross-sectional study design limits our ability to explore causal relationships directly. Although self-reported OA data is commonly used in large epidemiological studies, it is important to acknowledge its limitations compared to clinically assessed OA. it is known that OA prevalence varies depending on the definition used, with radiographic OA being more frequent than symptomatic OA. However, within individual joint sites, the prevalence estimates for self-reported OA and symptomatic OA tend to be similar, providing a reasonable approximation for large-scale studies like ours (53, 54). Despite this, since the osteoarthritis data is self-reported, recall bias among participants may have affected the accuracy of the included variables. Future studies with large-scale prospective samples are needed to better elucidate the potential causal relationships.

## 5 Conclusion

The results of our study demonstrate a significantly positive correlation between BRI and OA. This finding highlights the necessity of early intervention in individuals with elevated BRI to prevent the onset of OA. However, the results of the current cross-sectional study are insufficient to establish a direct causal relationship. Further large-scale prospective studies are needed to validate our results.

## Data Availability

The original contributions presented in the study are included in the article/supplementary material, further inquiries can be directed to the corresponding author.

## Footnotes

^1^http://www.empowerstats.com
